# Consideration of Pneumonectomy in the Era of Chemo-Immunotherapy in Resectable NSCLC

**DOI:** 10.1055/a-2564-2186

**Published:** 2025-04-21

**Authors:** Ibrahim Azar, Nikhil Vojjala, Richa Parikh, Hari B. Keshava, Misako Nagasaka

**Affiliations:** 1 Trinity Health IHA Medical Group Oakland Campus, Pontiac, United States; 2 Department of Medicine, Barbara Ann Karmanos Cancer Institute, Detroit, Michigan, United States; 3 Department of Surgery, University of California Irvine School of Medicine, Orange, California, United States; 4 Department of Oncology, University of California Irvine School of Medicine, Orange, California, United States

## Introduction

Recently, there has been increasing evidence for the use of neo-adjuvant chemoimmunotherapy in patients with resectable lung cancer. We aimed to analyze the pneumonectomy rates in the recently published phase 3 trials (including CM 816, KN 671, AEGEAN, and NEO-TORCH) 1 2 3 4 in patients with resectable NSCLC and compare them with historical data. In this paper, we highlight the changing total surgical and pneumonectomy rates in the era of chemo-immunotherapy in resectable nonsmall cell lung cancer (NSCLC) patients.

## Materials and Methods

We aimed to analyze the pneumonectomy rates in the recently published phase 3 trials in patients with resectable NSCLC and compare them with historical data. Pneumonectomy rates were collected from published or presented trials including CM 816, KN 671, AEGEAN, and NEO-TORCH. These trials enrolled patients with stage II and stage III disease except for CM 816 which also included stage IB patients. All four trials have used different immunotherapies.

## Results and Discussion

These trials report that neoadjuvant chemoimmunotherapy resulted in longer event-free survival (EFS) than chemotherapy in resectable NSCLC. The benefit of EFS was pronounced especially in the Stage IIIA group according to the CM-816 trial. 1 Historically, Felip et al have reported a total surgical resection rate of 91% with pneumonectomy rates of 23.2% in the preoperative chemotherapy arm of a landmark phase III study of neoadjuvant versus adjuvant chemotherapy in early-stage NSCLC. 5 Of note, this study included clinical stage IA, IB, II, or T3N1 disease. Similarly, Scagliotti et al reported a total surgical resection rate of 85.3% and pneumonectomy rates of 15.45% in the chemotherapy plus surgery arm of a phase III study of surgery alone or surgery plus preoperative chemotherapy. 6 In this study, stages IB to IIIA NSCLC were included. While challenges in surgical operability for lung cancer are easier to understand with patient comorbidities like advanced heart failure or extremely poor pulmonary function prohibiting an operation; surgical resectability of lung cancer is more ambiguous usually based on the particular surgeon the patient is seeing. In general, surgeons shy away from surgical resection in patients with bulky or multi-station N2 disease as an R0 resection is paramount. As all of the neoadjuvant chemoimmunotherapy studies enrolled stage IIIA patients who were evaluated to be amenable to surgery, direct comparisons to surveillance, epidemiology, and end results (SEER) data 7 cannot be performed. Analysis from the SEER database 7 shows that surgery was not recommended in 65% and was actually performed in only 25% of 22,558 stage IIIA NSCLC cases (10% did not undergo surgery due to patient preference, stage migration, or co-morbidities). This practice rests on the INT-0139 trial which showed no survival benefit from the addition of surgery to chemoradiation. 8

**Fig. 1:**
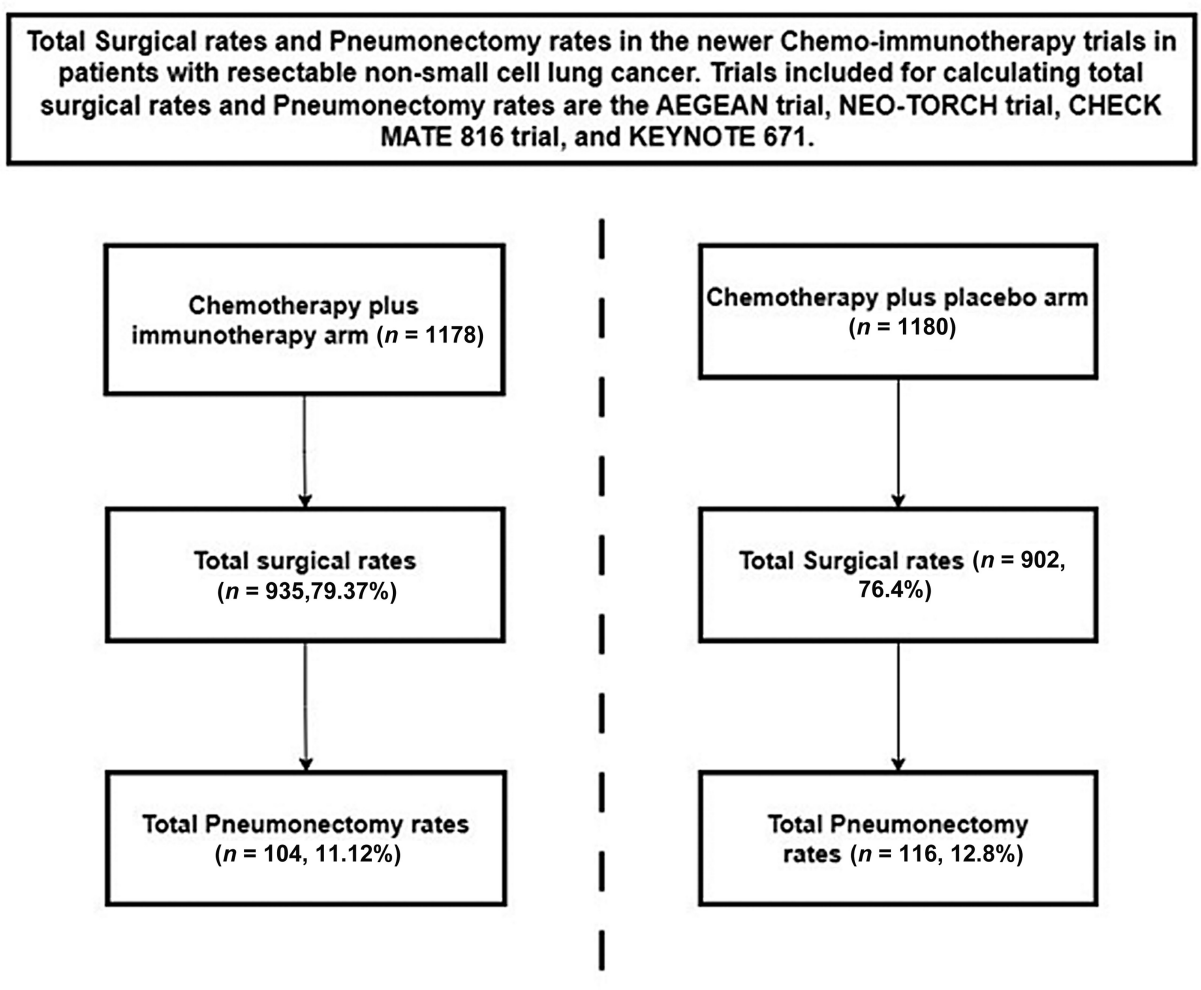
Flow diagram showing total surgical rates and pneumonectomy rates in the chemotherapy plus immunotherapy arm versus chemotherapy plus placebo arm.

When surgeons are faced with performing a pneumonectomy, an R0 resection is not the only consideration. There are acute changes in the postoperative setting which can be challenging, and there are long-term cardiovascular and respiratory changes that affect survival when compared with lobectomy. 9 While the overall pneumonectomy rates were lower in the four neoadjuvant chemo-immunotherapy studies (11.2% in the chemo-immunotherapy arm and 12.8% in the chemotherapy arm) compared with historical data, these numbers are not negligible ( Fig. 1 ). It is important to be reminded that the control arm of all four neoadjuvant chemo-immunotherapy trials was chemotherapy and not chemoradiation. This suggests that there may be a fair number of cases that would be left with reduced lung function when compelling data from the PACIFIC trial 10 has matured with an estimated 42.9% of patients randomly assigned to durvalumab remaining alive at 5 years versus 33.4% of patients randomly assigned to placebo remain alive and free of disease progression, establishing a new benchmark for the standard of care in this setting. However, the PACIFIC trial only included patients who were inoperable or not amenable to surgical resection. Adding to this, if patients are deemed to be unresectable after chemo-immunotherapy, they will be subjected to radiation therapy. Thus, a trial comparing overall survival and quality-of-life between definitive chemoradiation and neoadjuvant chemoimmunotherapy is warranted especially for those patients where pneumonectomy could be required for an R0 resection. Furthermore, Stage 3A is heterogeneous (T4N0, T3-T4N1, T1-T2N1, T1-T2N2). Reporting surgical/survival data by nodal status would also clarify the evolving role of surgery in the advent of immunotherapy in the nonmetastatic setting.
